# Establishment of the diagnostic and prognostic nomograms for pancreatic cancer with bone metastasis

**DOI:** 10.1038/s41598-022-21899-6

**Published:** 2022-10-27

**Authors:** Zongtai Liu, Haiyan Liu, Dalin Wang

**Affiliations:** 1 Department of Orthopedics, Affiliated Hospital of Beihua University, No.12 Jiefang Middle Road, Jilin, 132000 China; 2 Department of Orthopedics, Baicheng Central Hospital, Jilin, China

**Keywords:** Bone metastases, Bone cancer, Risk factors

## Abstract

Bone metastasis (BM) is rare in patients with pancreatic cancer (PC), but often neglected at the initial diagnosis and treatment. Bone metastasis is associated with a worse prognosis. This study was aimed to perform a large data analysis to determine the predictors and prognostic factors of BM in PC patients and to develop two nomograms to quantify the risks of BM and the prognosis of PC patients with BM. In the present study, we reviewed and collected the data of patients who were diagnosed as PC from 2010 to 2015 in the Surveillance, Epidemiology, and End Results (SEER) database. Univariate and multivariate logistic regression analyses were used together to screen and validate the risk factors for BM in PC patients. The independent prognostic factors for PC patients with BM were identified by Cox regression analysis. Finally, two nomograms were established via calibration curves, receiver operating characteristic (ROC) curve, and decision curve analysis (DCA). This study included 16,474 PC patients from the SEER database, and 226 of them were diagnosed with BM. The risk factors of BM for PC patients covered age, grade, T stage, N stage, tumor size, and primary site. The independent prognostic factors for PC patients with BM included age, race, grade, surgery, and lung metastasis. The AUC of the diagnostic nomogram was 0.728 in the training set and 0.690 in the testing set. In the prognostic nomogram, the AUC values of 6/12/18 month were 0.781/0.833/0.849 in the training set and 0.738/0.781/0.772 in the testing set. The calibration curve and DCA furtherly indicated the satisfactory clinical consistency of the nomograms. These nomograms could be accurate and personalized tools to predict the incidence of BM in PC patients and the prognosis of PC patients with BM. The nomograms can help clinicians make more personalized and effective treatment choices.

## Introduction

Pancreatic cancer (PC) is a malignancy with an extremely poor prognosis with the 5-year survival as low as 6%, ranking 7th in cancer mortality in England and Wales^1,2^. Due to the lack of effective and precise early diagnostic methods and non-specific symptoms, most patients are diagnosed in the late phase of the course. Only 10% of patients are capable to accept standard surgical excision which is still the only hope to treatPC patients at present^1,3^. The effect of distant metastases on prognosis is catastrophic. Distant metastases are responsible for nearly 90% of PC death^4^. In previous studies, 49.2% of patients had regional or distant metastases at initial diagnosis^5^. Distant metastases frequently occur in liver or peritoneum but rarely in the bone^6^. Bone metastasis (BM) is often an underappreciated site of PC metastasis, which has been reported that the incidence account 5% to 20% from all metastatic sites^7,8^.

Although the mechanism of BM in PC remains unclear, BM can lead to a series of complications such as hypercalcemia and pathological fracture, which further deterioratethe prognosis. Bone examinations, like X-rays, CT scans, magnetic resonance imaging, and positron emission topography (PET) scans, have been used to detect the presence of BM in PC. However, none of them have a superior detection rate^9^. It is reported that the association of BM and PC is higher in patients who have a primary tumor in the tail of the pancreas^10^. What’s more, there were many studies suggesting that some cytokines might play a non-negligible role in the invasion of pancreatic cancer in bone, like type I collagen (1CTP)^11^, interleukin-6 (IL-6), vascular endothelial growth factor (VEGF), and parathyroid hormone-related protein (PTHrP)^10^.

Nomogram has been accepted as a visual predictive tool based on the statistical regression models, which could help clinicians to make accurate decisions and promote the development of precision medicine^12^. The were some nomograms developed to evaluate the outcome and metastases of PC^13–16^. To our knowledge, there is no study focus on the predictive models for forecasting the BM in PC patients and the prognosis of PC patients with BM.

In the present study, we aimed to develop two nomograms that can predict the BM in PC patients and the overall survival (OS) of PC patients with BM based on the data of the Surveillance, Epidemiology, and End Results (SEER) database.

## Methods

### Study population

In the present study, we searched and obtained the clinical data from the Surveillance, Epidemiology, and End Results (SEER) database (Version 8.3.6). The inclusion criteria were as follows: (1) Patients were diagnosed histologically as PC between 2010 and 2015; (2) The primary tumor site was pancreas; (3) The information of clinical and demographic features needed for the study were intact and accessible. In addition, the patients diagnosed with autopsies or death certificates were excluded from the study. Finally, there were 16,474 patients included in the cohort to study the risk factors of BM in PC patients and to establish a predictive nomogram. As for the exploration of prognostic factors for PC patients with BM, a total of 226 patients were enrolled in the cohort. As this study did not involve human subjects or personal privacy, the information consent from patients and ethical approval were not required.

### Data collection

In the present study, seven variables were used to identify the risk factors of BM in PC, including sex, age, race, primary site, grade, T stage (AJCC 7th), N stage (AJCC 7th), and tumor size. For the study about the prognostic factors for PC patients with BM, the information of treatment variables and metastasis date were also added to the cohort, including surgery, chemotherapy, radiotherapy, liver metastasis, lung metastasis, and brain metastasis. In the prognostic study, overall survival (OS) was identified as the primary outcome, which was defined as the survival time from diagnosis to death due to any cause.

### Statistical analysis

All statistic analyses in the present study were performed with SPSS 25.0, R software (version 4.0.1), and X-tile. The univariate and multivariate analyses were used to identify the independent risk factors of BM and prognostic factors of PC patients with BM. In the present study, a P-value < 0.05 was considered as significant difference. The predictive and prognostic nomograms were established by the R packages “rms” and “regplot”.

In the study of the risk factor of BM from PC, univariate and multivariate logistic were applied to identify the risk factor of BM. Besides, we also performed the receiver operating characteristic (ROC) and area under the curve (AUC) to estimate the discrimination of the model. The calibration curve and decision curve analysis (DCA) were developed to further estimate the performance of the models. As for the prognosis of PC patients with BM, the time-dependent ROC, calibration, and DCA were also caculated. Furthermore, according to the cut-off value of the total nomogram points, patients were divided into two risk levels and the Kaplan–Meier (K–M) survival curve with a log-rank test was generated to verify the prognostic value of the nomogram. In addition, ROC curves or time-dependent ROC curves of all independent variables were also generated to compare the AUCs of the nomogram with all independent variables.

## Results

### The characteristics of the population in the diagnostic cohort

In the present study, a total of 16,474 patients were included in the cohort, and 226 (1.40%) of them were diagnosed as BM at diagnosis. Meanwhile, 11,530 (70%) patients were divided into the training set and others 4944 (30%) were into the validation set. The baseline of the 16,474 patients was shown in Table 1. In the training set, the majority patients were White in race distribution (80.16%) and had lesion in pancres head (65.72%). There were no difference in clinical characteristics, like age, race, grade, T/N stage, sites, tumor size, and bone mastasis, between two cohorts (*P* > 0.05).

**Table 1:** Clinical characteristics of 16,474 pancreatic cancer patients.

	Total set (n = 16,474)	Training set (n = 11,530)	Validation set (n = 4944)	Χ^2^	P
**Sex**				2.497	0.114
Female	7909	5489	2420		
Male	8565	6041	2524		
**Age, years**				1.423	0.233
≤ 66	8024	5651	2373		
> 66	8450	5879	2571		
**Race**				0.921	0.630
Black	1845	1302	543		
White	13,205	9245	3960		
Other	1424	983	441		
**Primary site**				4.173	0.383
Head	10,068	7041	3027		
Body	2105	1466	639		
Tail	2872	2046	826		
Other	274	185	89		
Overlapping lesion	1155	792	363		
**Grade**				2.603	0.457
I	3480	2398	1082		
II	6730	4732	1998		
III	5863	4115	1748		
IV	401	285	116		
**T**				1.113	0.774
T1	1523	1057	466		
T2	3191	2218	949		
T3	9534	6618	2855		
T4	2326	1637	674		
**N**				< 0.001	0.995
N0	8393	5874	2519		
N1	8081	5656	2425		
**Tumor size, mm**				1.738	0.187
≤ 34	7625	5298	2327		
> 34	8849	6232	2617		
**Bone metastasis**				0.312	0.576
No	16,248	11,368	4880		
Yes	226	162	64		

### Risk factors of BM in PC patients

The univariate logistic analyses were performed to identify the risk factors of BM in PC patients. As shown in Table 2, the results showed that age, primary site, tumor size, grade, T stage, and N stage were related to BM. After that, variables were incorporated into the multivariate logistic analysis, all the six factors were independent risk factors of BM in newly diagnosed PC patients.

**Table 2:** Univariate and multivariate logistic analyses of BM in PC patients.

	Univariate analysis				Multivariate analysis			
	OR	95%CI		*P*	OR	95%CI		*P*
**Sex**								
Female	Reference							
Male	0.978	0.717–1.334		0.889				
**Age, years**								
≤ 66	Reference				Reference			
> 66	0.640	0.467–0.878		0.006	0.656	0.476–0.904		0.010
**Race**								
Black	Reference							
Other	1.180	0.599–2.326		0.632				
White	1.001	0.609–1.646		0.995				
**Primary site**								
Head	Reference				Reference			
Body	1.767	1.133–2.755		0.012	1.435	0.908-2.267		0.112
Tail	1.877	1.274–2.767		0.001	1.786	1.191-2.679		0.005
Other	2.081	0.753–5.752		0.158	2.293	0.816-6.443		0.115
Overlapping lesion	2.065	1.213–3.517		0.008	1.485	0.860-2.564		0.156
**Grade**								
I	Reference				Reference			
II	1.356	0.819–2.244		0.237	1.278	0.761–2.148		0.354
III	1.959	1.200–3.198		0.007	1.689	1.014–2.811		0.044
IV	6.288	3.204–12.344		< 0.001	4.759	2.381–9.514		< 0.001
**T**								
T1	Reference							
T2	6.946	2.150–22.440		0.001	3.994	1.188–13.433		0.025
T3	3.485	1.093–11.108		0.035	1.907	0.562–6.474		0.301
T4	11.298	3.517–36.292		< 0.001	5.336	1.551–18.353		0.008
**N**								
N0	Reference				Reference			
N1	1.371	1.003–1.875		0.048	1.494	1.073–2.082		0.018
**Tumor size, mm**								
≤ 34	Reference				Reference			
> 34	2.625	1.833–3.758		< 0.001	1.758	1.199–2.578		0.004

OR: Odds ratio; CI: Confidence interval; BM: Bone metastasis; PC: Pancreatic Cancer.

### Development and validation of the diagnostic nomogram

**Figure 1:**
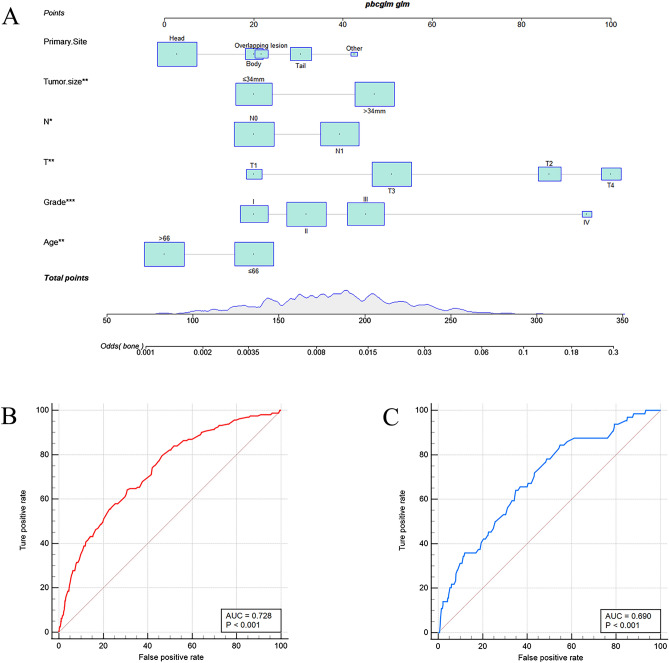
The nomogram incorporating six clinical variables for predicting the risk of BM in PC patients (**A**); The receiver operating characteristic curve of nomogram in the training set (**B**); The receiver operating characteristic curve of nomogram in the validation set (**C**).

**Figure.2:**
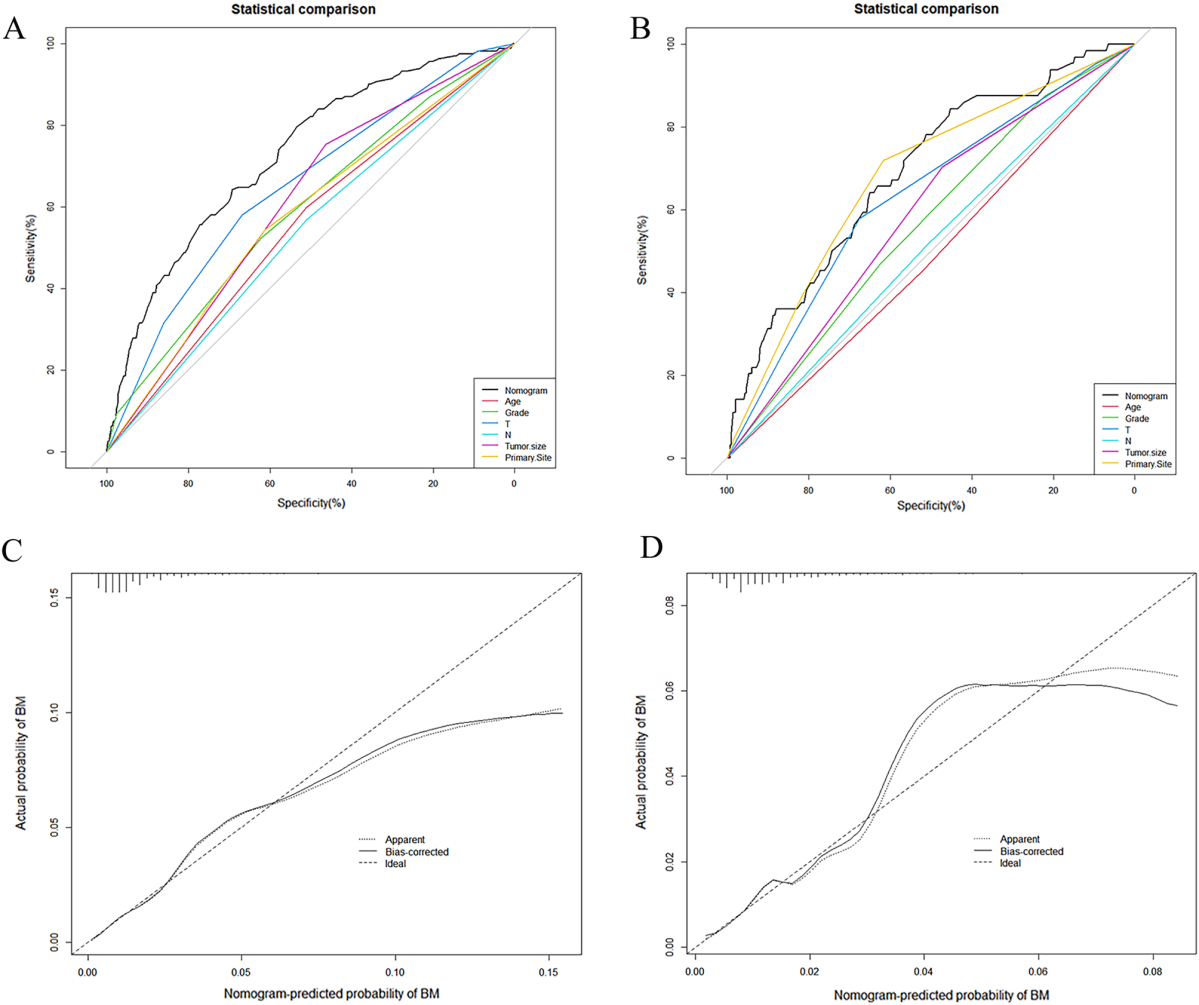
Comparison of the values of area under the curve between nomogram and single independent risk factors in the training set (**A**) and validation set (**B**); The calibration curve of nomogram in the training set (**C**) and validation set (**D**).

**Figure 3:**
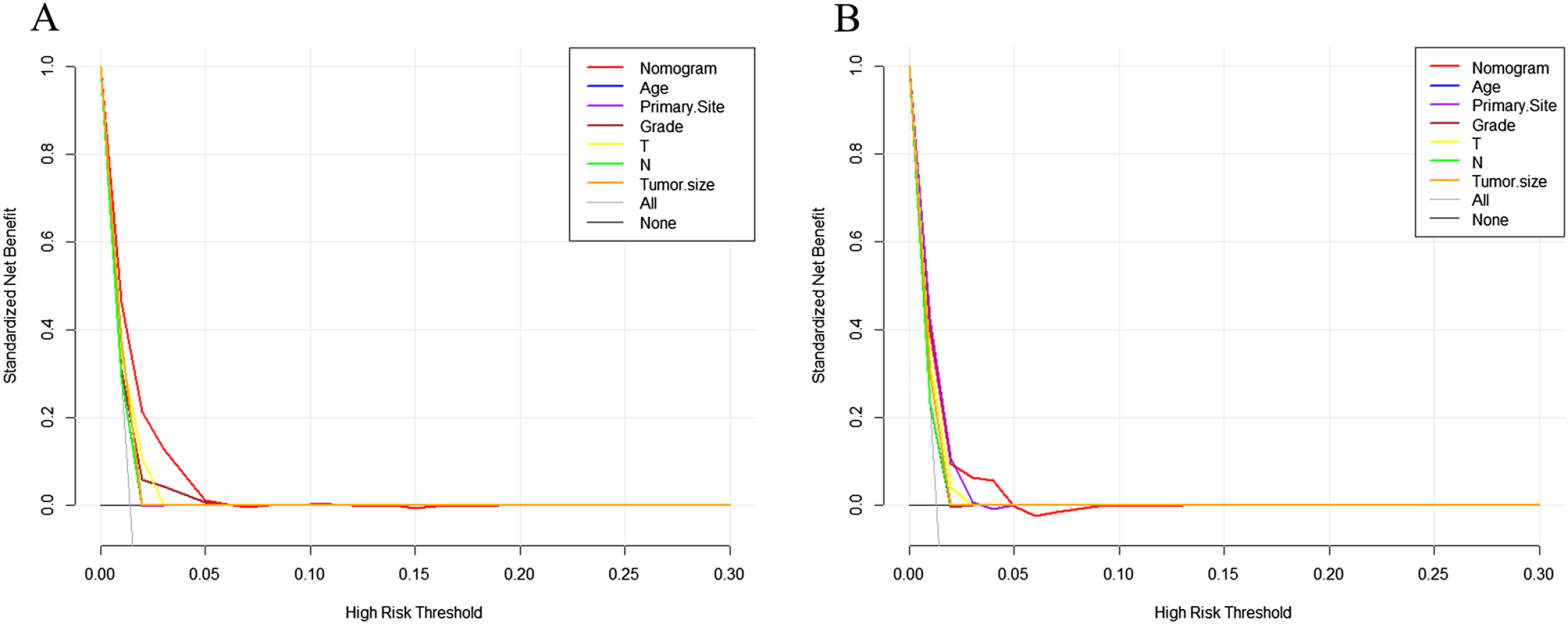
Comparison of decision curve analysis between the diagnostic nomogram and single independent factors in the training set (**A**) and validation set (**B**).

Based on the six independent risk factors, a diagnostic nomogram was established (Fig. 1A). The ROC curves of the training set and validation set were generated, and the corresponding AUC values were 0.728 and 0.690 in the training set and validation set, respectively (Fig. 1B,C). Furthermore, ROC curves comparisons against all other risk factors were also generated (Fig. 2A,B). The results showed that the AUC of the nomogram was higher than any other single factors, both in the training set and validation set. More importantly, the calibration curves of both sets showed high consistency between the observed and predicted results (Fig. 2C,D). Finally, the DCA indicated the nomogram could be a more effective tool than other single factors in clinical practice (Fig. 3A,B).

### Prognostic factors for PC patients with BM

A total of 226 were eventually enrolled in the prognostic analyses. Similarly, 159 (70%) patients were randomly assigned into to the training set and others 67 (40%) were assigned into the validation set. The demographic and clinical information of the 226 patients was shown in Table 3. There were no difference in clinical characteristics between two cohorts, like age, race, grade, T/N stage, sites, tumor size, surgery, radiotherapy, and chemotherapy (*P* > 0.05).

**Table 3:** Clinical characteristics of 226 PC patients with BM.

	Total (n = 226)	Training set (n = 159)	Validation set (n = 67)	Χ^2^	P
**Sex**				1.581	0.209
Female	109	81	28		
Male	117	78	39		
**Age, years**				1.164	0.559
< 56	44	32	12		
56–67	89	59	30		
> 67	93	68	25		
**Race**				2.908	0.234
Black	25	15	10		
Other	17	10	7		
White	184	134	50		
**Grade**				0.360	0.948
I	29	20	9		
II	82	57	25		
III	98	69	29		
IV	17	13	4		
**Primary site**				1.217	0.875
Head	92	62	30		
Body	40	30	10		
Tail	62	43	19		
Other	7	5	2		
Overlapping lesion	25	19	6		
**T**				2.584	0.460
T1	6	3	3		
T2	64	46	18		
T3	89	66	23		
T4	67	44	23		
**N**				0.324	0.569
N0	101	73	28		
N1	125	86	39		
**Tumor size, mm**				5.480	0.065
< 41	99	62	37		
41–114	103	80	23		
> 114	24	17	7		
**Surgery**				0.151	0.698
No	205	145	60		
Yes	21	14	7		
**Radiotherapy**				0.875	0.350
No	168	121	47		
Yes	58	38	20		
**Chemotherapy**				0.029	0.866
No	93	66	27		
Yes	133	93	40		
**Brain metastasis**				< 0.001	> 0.999
No	216	152	64		
Yes	10	7	3		
**Liver metastasis**				1.843	0.175
No	73	47	26		
Yes	153	112	41		
**Lung metastasis**				0.085	0.771
No	152	106	46		
Yes	74	53	21		

To identify the independent prognostic factors for PC patients with BM, the univariate and multivariate Cox analyses were performed. As shown in Table 4, the OS-related factors included age, race, grade, surgery, lung metastasis, and tumor size. Finally, the multivariate Cox analysis revealed that age, race, grade, surgery, and lung metastasis were independent prognostic risk factors of PC patients with BM.

**Table 4:** Univariate and multivariate Cox analyses of PC patients with BM.

	Univariate analysis				Multivariate analysis			
	HR	95%CI		P	HR	95%CI		P
**Sex**								
Female	Reference							
Male	0.987	0.715–0.362		0.935				
**Age, years**								
< 56	Reference				Reference			
56–67	1.574	0.991–2.501		0.055	1.556	0.950–2.548		0.079
> 67	1.710	1.086–2.693		0.021	2.283	1.408–3.701		0.001
**Race**								
Black	Reference				Reference			
Other	0.801	0.358–1.793		0.590	0.544	0.233–1.270		0.159
White	0.469	0.271–0.811		0.007	0.465	0.259–0.834		0.010
**Grade**								
I	Reference				Reference			
II	2.923	1.649–5.182		< 0.001	3.113	1.732–5.594		< 0.001
III	4.567	2.549–8.182		< 0.001	5.110	2.799–9.328		< 0.001
IV	3.582	1.685–7.615		0.001	4.100	1.854–9.069		< 0.001
**Primary site**								
Head	Reference							
Body	1.018	0.648–1.598		0.938				
Tail	1.233	0.826–1.839		0.306				
Other	1.736	0.692–4.354		0.240				
Overlapping lesion	1.326	0.788–2.231		0.289				
**T**								
T1	Reference							
T2	1.182	0.366–3.819		0.780				
T3	0.978	0.306–3.126		0.970				
T4	0.974	0.300–3.161		0.965				
**N**								
N0	Reference							
N1	1.335	0.965–1.847		0.081				
**Tumor size, mm**								
< 41	Reference				Reference			
41–114	1.522	1.077–2.151		0.017	1.382	0.487–3.918		0.543
> 114	2.851	1.628–4.993		< 0.001	2.608	0.935–7.277		0.067
**Surgery**								
No	Reference				Reference			
Yes	0.379	0.208–0.690		0.002	0.410	0.221–0.762		0.005
**Radiation**								
No	Reference							
Yes	0.745	0.510–1.089		0.128				
**Chemotherapy**								
No	Reference							
Yes	0.820	0.586–1.146		0.246				
**Brain metastasis**								
No	Reference							
Yes	1.797	0.833–3.873		0.135				
**Liver metastasis**								
No	Reference							
Yes	1.329	0.934–1.892		0.114				
**Lung metastasis**								
No	Reference				Reference			
Yes	1.625	1.150–2.298		0.006	1.466	1.012–2.123		0.043

*HR* Hazard ratio, *CI* Confidence interval, *BM* Bone metastasis, *PC* Pancreatic Cancer.

### Development and validation of the prognostic nomogram

**Figure 4:**
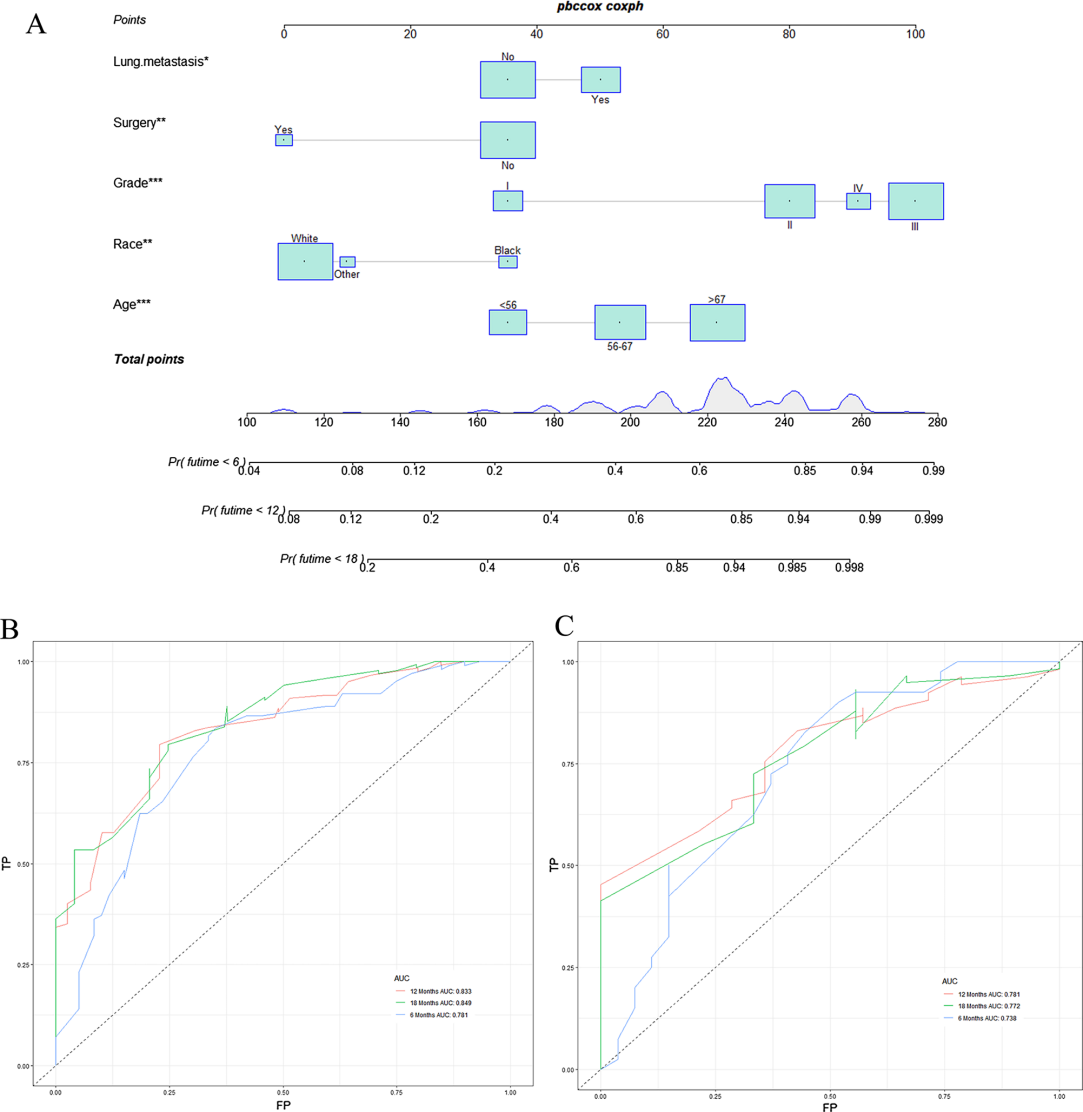
Prognostic nomogram for predicting the overall survival of PC patients with BM (**A**); Two time-dependent receiver characteristic curves to show the discrimination of prognostic nomogram in training set (**B**) and validation set (**C**).

**Figure 5:**
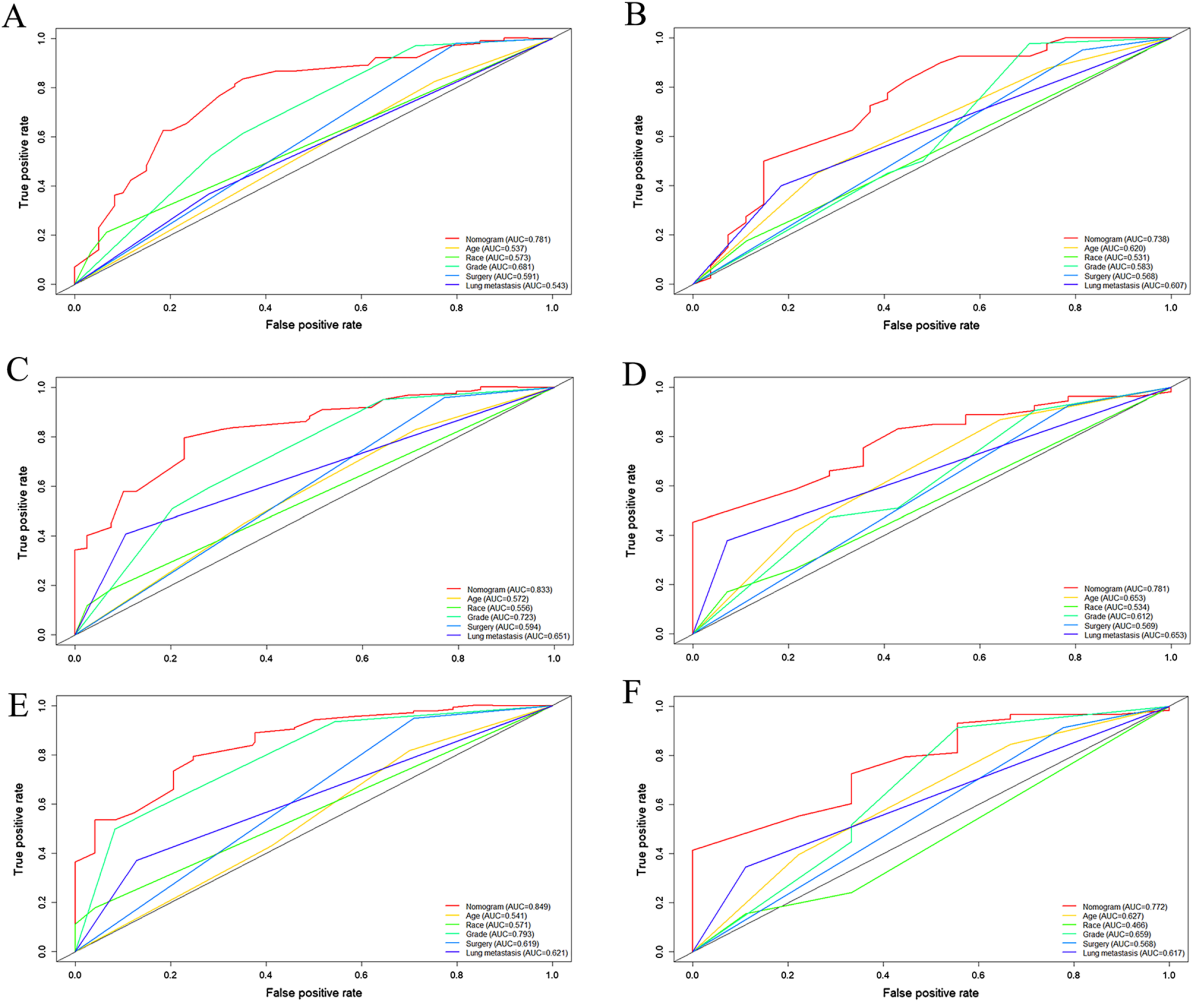
Comparison of the values of area under the curve between nomogram and single independent factors. 6-month in the training set (**A**); 12-month survival in the training set (**C**); 18-month survival in the training set (**E**); 6-month survival in the validation set (**B**); 12-month survival in the validation set (**D**); 18-month survival in the validation set (**F**).

**Figure 6:**
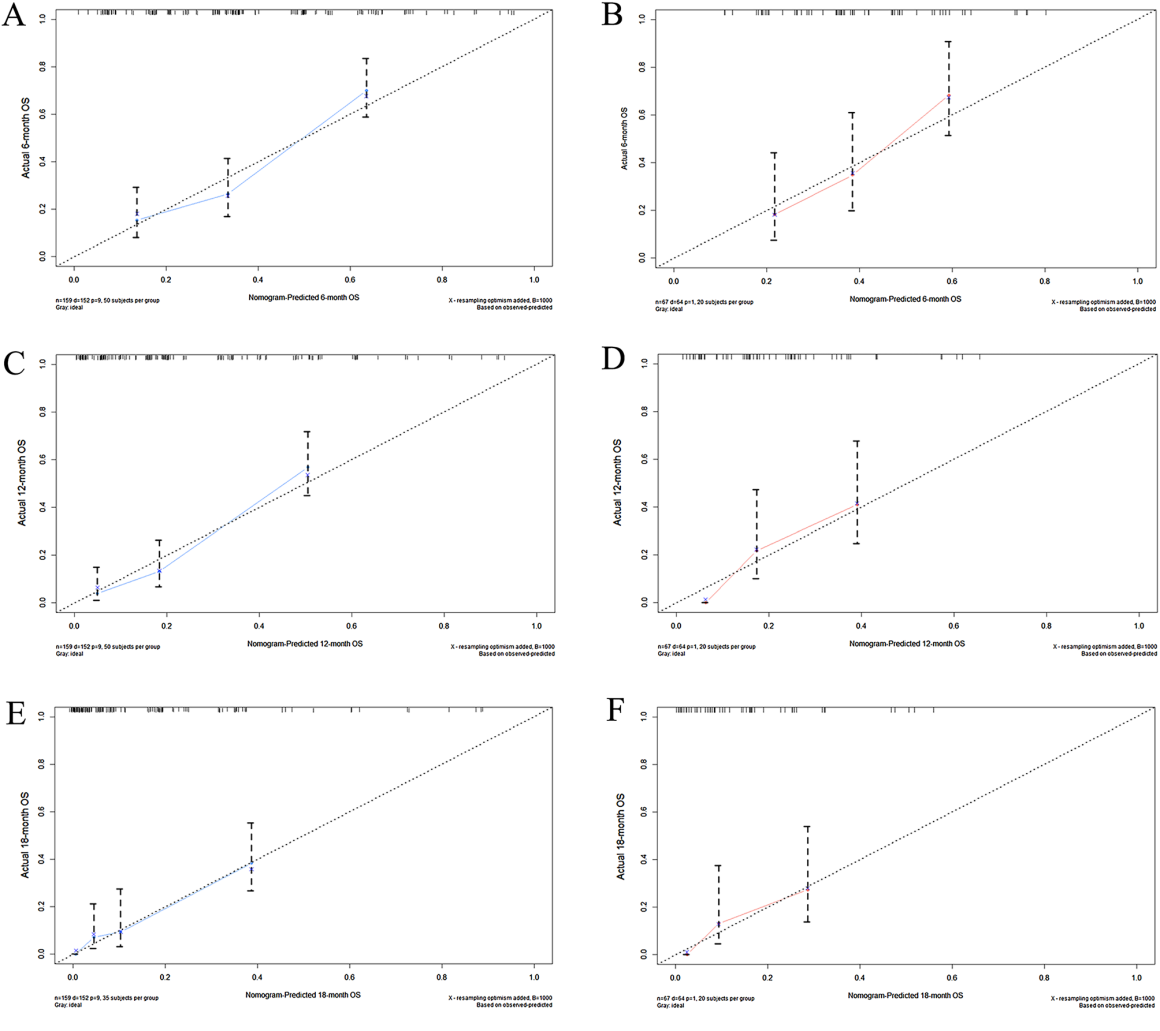
Calibration curves of 6-month in the training set (**A**); 12-month survival in the training set (**C**); 18-years survival in the training set (**E**); 6-month survival in the validation set (**B**); 12-month survival in the validation set (**D**); 18-years survival in the validation set (**F**).

**Figure 7:**
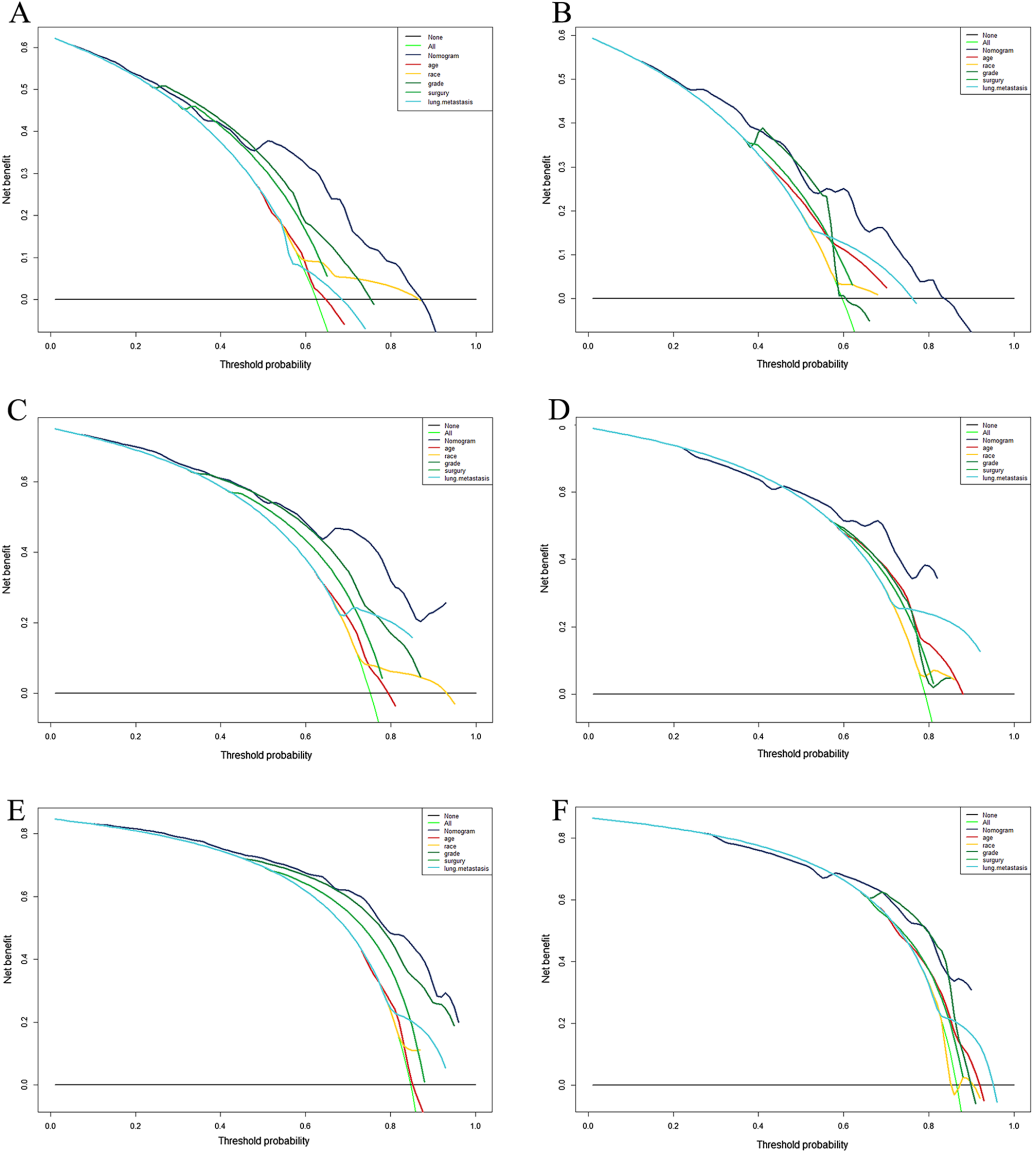
Comparison of decision curve analysis between the prognostic nomogram and single independent factors. 6-month survival in the training set (**A**); 12-month survival in the training set (**C**); 18-years survival in the training set (**E**); 6-month survival in the validation set (**B**); 12-month survival in the validation set (**D)**; 18-years survival in the validation set (**F**).

A prognostic nomogram was established based on the five independent prognostic risk factors (Fig. 4A). The AUC values for the nomogram predicting 6-, 12-, and 18-month OS were 0.781/0.833/0.849 in the training set and 0.738/0.781/0.772 in the validation set, respectively (Fig. 4B,C). In the comparison with other single factors, the nomogram had higher AUCs at 6-, 12-, and 18-month than all other single factors for each set (Fig. 5A–F). Moreover, the calibration curves showed that the nomogram-predicted OS were in satisfactory agreement with actual OS at 6-, 12-, 18-month in both sets (Fig. 6A–F). Eventually, the DCA curves of both sets indicated that the nomogram had better predictive performance than single factors in predicting OS of PC patients with BM (Fig. 7A–F). As a whole, the nomogram can be served as a reliable tool for predicting the OS for PC patients with BM and help clinicians make more personal medical decisions.

### Stratification of risk groups

**Figure 8:**
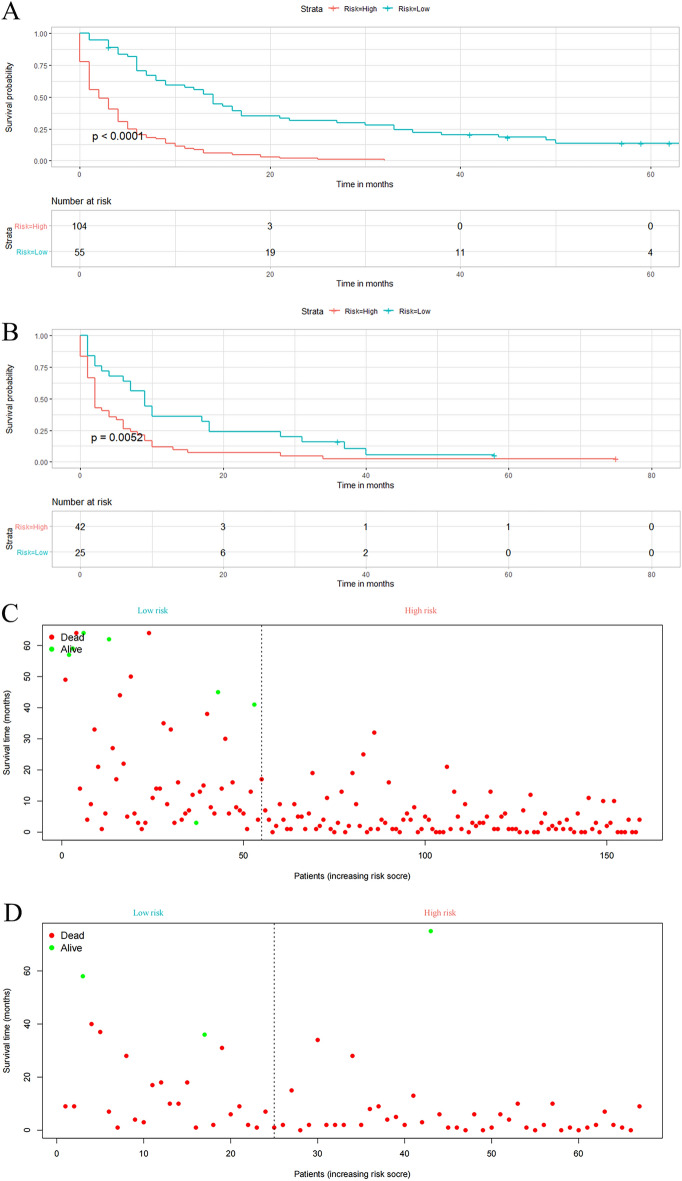
Risk stratification for PC patients with BM. Survival curve of two risk groups in the training set (**A**) and validation set (**B**); a scatter diagram to show the survival status of patients in the training set (**C**) and validation set (**D**).

According to the cut-off value of the risk score in training set via the X-tile program, patients were divided into two groups, low-risk group (score ≤ 208) and high-risk group (score > 209). The K–M survival curves with a log-rank test were established and it was not difficult to find that the prognoses among the two groups were significantly different (*P* < 0.0001, Fig. 8A). The same cut-off values were also used in the validation set. The K–M survival curves of validation set showed that the high-risk group had a worse prognosis than low-risk groups (*P* = 0.0052, Fig. 8B). In addition, to show the difference between the groups more intuitively, two scatter diagrams were plotted (Fig. 8C,D). It was clear to find that the patients’ survival time gets shorter and shorter as the risk score increases.

### Ethics approval and consent to participate

We received permission to access the research data file in the SEER program from the National Cancer Institute, US (reference number 15260-Nov2018). Approval was waived by the local ethics committee, as SEER data is publicly available and de-identified.

## Discussion

PC remains one of the most deadly disease of all cancers. Autopsy series have shown that nearly 90% of cases of PC were complicated with distant metastases^17^. According to the previous studies, patients with distant metastases have a lower 5-year survival rate compared with patients without metastases^18^. Bone metastases in PC are considered to be relatively rare compared to liver or peritoneal metastases^8^. However, with the development of PC incidence and detection techniques, more and more patients with BM will be found, so attention should be paid to these patients. In the present study, tumor size, grade, T stage, N stage, age, and primary site were independent diagnostic factors of BM for PC patients. In addition, surgery performed, grade, lung metastases, race, and age were identified as independent prognostic factors for PC patients with BM. Based on these risk factors, we established two nomograms to predict the risk of BM for PC patients and the 6-, 12-, 18-month OS of PC patients with BM, respectively. Both nomograms showed good consistency between the predictive results and the actual result. The use of the diagnostic and prognostic nomograms can benefit both the clinicians and individual patients.

There is no doubt that improving the clinical skills and means to detect early-stage of PC and metastases plays a vital role in prolonging the survivals of PC patients. However, despite considerable efforts, the pathogenesis and specific molecular mechanism of BM in PC remain unclear. Therefore, genetic screening and tumor biomarker screening are still difficult to be effectively applied in clinic at present. Although some previous studies have focused on the risk factors of PC, to our knowledge, there is no study focusing on the risk factors associated with BM in PC. In our results, higher T stage, N stage, and larger tumor size were associated with a higher risk of BM, similar to other kinds of tumors^19–21^. Notably, younger patients (≤ 66 years) were more likely to develop BM than older patients. This may be due to younger patients with a family history of PC more probably. Previous studies have suggested that PC patients with a family history may have an earlier onset^22,23^. Wang et al. also reported that the relatives of patients with PC had a higher risk of dying from cancers at other sites^24^. In addition, our results showed that patients with T2 stage had a higher risk of BM than patients with T3 stage. The phenomenon may be because that patients with T3 stage have more obvious clinical symptoms due to the invasion of surrounding organs and tissues so that patients are more proactive in the disease examination^25^.

Early-stage PC is usually clinically silent, and most patients who developed symptoms may have missed the best time for treatment. In our study, surgery performed remains an important prognostic factor. With the development of surgical techniques, such as laparoscopy, more and more patients who could not tolerate surgery in the past are able to receive surgical treatment. However, due to the complexity and serious complications of the surgery, the prognosis of patients who received surgery in hospitals at different ranks have a big difference^26^. A previous population-based study indicated an approximately 50% reduction in the risk of postoperative mortality in the high-volume hospital versus low-volume hospitals between 2009 and 2014^27^. In addition, the nihilistic and skeptical attitude among patients and the concerns about the safety and efficacy of surgery of clinicians may also contribute to the low resection rates^3^. In our results, the difference in prognosis caused by racial difference is also noteworthy. Some past studies have suggested that the black patients had a worse prognosis due to the lower overall economic status or worse lifestyles habits such as a higher rate of smoking, alcohol consumption, and diabetes^28,29^. However, Nipp et al. indicated that even adjusting the potential confounding sociodemographic and clinical factors, the black PC patients also have a worse survival outcome^30^. More detailed prospective studies and genetic related studies will further explain these phenomena in the future. Moreover, older age also means worse prognosis, which is consistent with some previous studies^31–33^. Although chemotherapy is routinely used for the treatment of metastatic PC, it was not an independent prognostic factor in our results. This may be because the improvement of the prognosis of BM of PC patients by existing chemotherapy plans is not obvious. The lack of detailed chemotherapy information in the SEER database may also contribute to this phenomenon. Some new chemotherapy plans in recent years may further expand the impact of chemotherapy on the prognosis of PC patients with BM^34,35^. Neoadjuvant chemotherapy and immunotherapy also provide a new insight for the treatment of patients^36,37^. Interestingly, we found that the presence or absence of lung metastases also influenced the prognosis of PC patients with BM. This may be because some biomarkers such as IF-6 may be expressed at the same high level in lung metastasis and BM, so that lung metastasis can reflect the severity of BM to some extent and affect the prognosis^10,38,39^. Further research may provide a direction for future research on blocking drugs.

### Limitations

To our knowledge, this study is the first diagnostic and prognostic model to predict the risks and the prognosis of BM in PC based on a large population. However, there were still some limitations in the present study. First, the limited number of patients may make conclusions less conclusive. Second, the inevitable selection bias associated with retrospective studies. Third, due to the limited factors included in the SEER database, some possible risk factors were not included in the study, such as dietary habits, diabetes, and specific treatment plans. Finally, the more advanced version 8th AJCC staging could not be used due to the data logging limitations and the inability to efficiently convert. Despite these limitations, the present study offers the possibility of predicting the risk of BM in PC and the prognosis of PC patients with BM.

## Conclusion

The present showed that tumor size, grade, T stage, N stage, age, and primary site were independent diagnostic factors of BM for PC patients. As for PC patients with BM, surgery performed, grade, lung metastases, race, and age were independent prognostic factors. Two nomograms were established and proved to have satisfactory performance. These convenient and visual tools can be used in risk assessment and prognostic prediction for BM in PC.

## Data Availability

The datasets generated and/or analyzed during the current study are available in the SEER database (https://seer.cancer.gov/).

## Abbreviations

BM: Bone metastasis
PC: Pancreatic cancer
SEER: Surveillance, Epidemiology, and End Results
ROC: Receiver operating characteristic
DCA: Decision curve analysis
1CTP: Type I collagen
IL-6: Interleukin-6
VEGF: Vascular endothelial growth factor
PTHrP: Parathyroid hormone-related protein
OS: Overall survival
AUC: Area under the curve
